# A Multistep Workflow to Evaluate Newly Generated iPSCs and Their Ability to Generate Different Cell Types

**DOI:** 10.3390/mps4030050

**Published:** 2021-07-19

**Authors:** Carol X.-Q. Chen, Narges Abdian, Gilles Maussion, Rhalena A. Thomas, Iveta Demirova, Eddie Cai, Mahdieh Tabatabaei, Lenore K. Beitel, Jason Karamchandani, Edward A. Fon, Thomas M. Durcan

**Affiliations:** 1 The Neuro’s Early Drug Discovery Unit (EDDU), McGill University, 3801 University Street, Montreal, QC H3A 2B4, Canada; xiuqing.chen@mcgill.ca (C.X.-Q.C.); narges.abdian@mcgill.ca (N.A.); gilles.maussion@mcgill.ca (G.M.); rhalena.thomas@mcgill.ca (R.A.T.); iveta.demirova@mail.mcgill.ca (I.D.); eddie.cai@mail.mcgill.ca (E.C.); lenore.beitel@mcgill.ca (L.K.B.); ted.fon@mcgill.ca (E.A.F.); 2 The Neuro’s Clinical Biological Imaging and Genetic Repository (C-BIG), McGill University, 3801 University Street, Montreal, QC H3A 2B4, Canada; ma.tabatabaei@mcgill.ca (M.T.); jason.karamchandani@mcgill.ca (J.K.)

**Keywords:** human-induced pluripotent stem cells, quality control, genomic integrity, trilineage differentiation, neural progenitor cells, cortical neurons

## Abstract

Induced pluripotent stem cells (iPSCs) derived from human somatic cells have created new opportunities to generate disease-relevant cells. Thus, as the use of patient-derived stem cells has become more widespread, having a workflow to monitor each line is critical. This ensures iPSCs pass a suite of quality-control measures, promoting reproducibility across experiments and between labs. With this in mind, we established a multistep workflow to assess our newly generated iPSCs. Our workflow tests four benchmarks: cell growth, genomic stability, pluripotency, and the ability to form the three germline layers. We also outline a simple test for assessing cell growth and highlight the need to compare different growth media. Genomic integrity in the human iPSCs is analyzed by G-band karyotyping and a qPCR-based test for the detection of common karyotypic abnormalities. Finally, we confirm that the iPSC lines can differentiate into a given cell type, using a trilineage assay, and later confirm that each iPSC can be differentiated into one cell type of interest, with a focus on the generation of cortical neurons. Taken together, we present a multistep quality-control workflow to evaluate newly generated iPSCs and detail the findings on these lines as they are tested within the workflow.

## 1. Introduction

**Figure 1:**
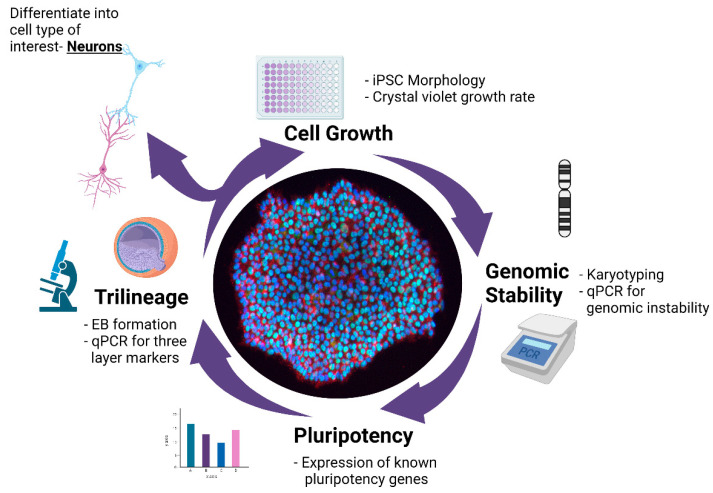
Multistep workflow for phenotyping of hiPSCs. Schematic representation of a multistep QC workflow for newly generated iPSCs to monitor morphology and proliferation, genomic integrity, pluripotency, and ability to form cells of the three germ layers.

Human pluripotent stem cells can give rise to almost any cell type when exposed to the appropriate developmental cues, holding enormous potential for tissue engineering, regenerative medicine, and disease modeling. The growing number of iPSC lines and National Institutes of Health (NIH)-registered human embryonic stem cell (ESC) lines ensures that patient-derived pluripotent stem cells are now readily available to researchers, helping to accelerate our understanding of biology and disease and the development of therapies across disease areas [1]. These advances underscore the need for iPSC quality standards that are sufficiently stringent to ensure that findings can be compared and results reproduced across laboratories [2,3]. Here, we provide a multistep workflow (Figure 1) that is scalable and targeted for iPSCs to be used in research applications. Each test is simple and straightforward, and the modular nature of the workflow means additional tests can be added if needed, based on the availability of resources. However, by assaying growth rate, genomic instability, pluripotency, and differentiation for a cell line, we can establish how well it performs and if it can used to generate the cells needed for downstream applications. Previous studies have used teratoma assays to demonstrate whether a line could generate all three cell types, but by combining a trilineage test with established protocols to generate a given cell type of interest, we not only can demonstrate that a line can generate all three lineages, but we can do it in a more cost-effective manner, and one which does not require animals, as the work only requires the cells themselves and the requisite reagents required in the protocol.

Since the advent of iPSC technology, several media formulations have been developed to grow and maintain iPSCs, although the growth rate of a line can vary based on the media used [4,5]. Thus, depending on the media used, iPSC growth can vary, making it imperative that the optimal growth media and maintenance conditions be defined before working with any new cell line and to reduce variation between lines [5,6,7,8]. In parallel with growth rate, monitoring the genomic integrity of stem cells is critical to assess each line before they can be used in research or clinical applications. Common alterations arise in the genome during the iPSC reprogramming process, from extensive culturing [9] or as a result of off-target CRISPR/Cas9 genome editing [10,11]. These alterations can include (1) large chromosomal duplications or rearrangements, assessed by karyotyping [12,13,14], (2) copy number variations assessed by qPCR, and (3) small genomic insertions and deletions or single-nucleotide variants (SNPs), detected by whole genome sequencing [15]. When present, these genomic abnormalities often alter the biological properties of hiPSC-derived models. Each benchmark for evaluating iPSCs is important, but has its own strengths and limitations in terms of sensitivity, cost, and time. For instance, karyotyping will detect all large chromosomal changes, but it is slow, and small genomic alternations are not detected. Copy number variations can be detected by qPCR, however only known common variants are detected. For detecting SNPs, genome sequencing is the best approach, but it comes at a higher cost and requires extensive computational analysis [12,13,14]. Building on our assays for iPSC growth rate and genomic alterations, in this paper, we present a multistep workflow to combine the aforementioned tests with additional checks for pluripotency and the differentiation capacity of the cell line. We evaluated the pluripotency of our lines by examining their ability to form embryoid bodies (EBs), to differentiate into each of the three germ layers, and their potential to form cortical neurons. We focused on cortical neurons, as methods to generate these neurons from iPSCs are well-established, and represent a cell type broadly studied across disease areas of the brain, from Alzheimer’s disease to neurodevelopmental disorders [16,17]. We applied these tests to ten newly generated hiPSC lines derived from healthy individuals and two commercial lines. Taken together, we developed a multistep, QC (quality control) workflow (Figure 1) to assess the growth rate, genomic stability, pluripotency, and capacity of each line to differentiate into a given trilineage or cell type for downstream applications.

## 2. Materials and Methods

### 2.1. Cell Lines

The lines used were: H9 hESC (Wicell), NCRM1 (NIH), KYOU-DXR0109B (ATCC), AIW002-02, AJC001-5, AJG001-C4 (these three lines are from the same donor but reprogrammed using different methods or from different cell types), AJD002-3, TD03 (these two lines are from the same donor but reprogrammed with different methods), TD2, TD10, TD22, 3448, and 3450. The complete profiles of the iPSCs are listed in Table 1. The use of iPSCs and stem cells in this research was approved by the McGill University Health Centre Research Ethics Board (DURCAN_IPSC/2019-5374).

### 2.2. iPSC Reprogramming

PBMCs and skin fibroblast cells were obtained through the C-BIG Repository at the Montreal Neurological Institute (The Neuro). AIW002-02, AJC001-5, and AJD002-3 were generated using the CytoTune™-iPS 2.0 Sendai Reprogramming Kit (iPSQuebec Platform, Laval University) and TD02, TD03, TD10, and TD22 through episomal reprogramming (Axol Biosciences, Cambridge, UK). AJG001-C4, 3448, and 3450 were generated in-house by episomal reprogramming [18]. Briefly, PBMCs were cultured for 6 days, and 2~3 × 10^6^ cells were nucleofected with episomal plasmids (pEV-OCT4-2A-SOX2, pEV-MYC, pEV-KLF4, and pEV-BC-XL, a generous gift from Dr. XB Zhang, Loma Linda University). The transfected PBMCs were plated on mitomycin C-treated mouse embryonic fibroblasts cultured in KnockOut DMEM/F12 supplement, with 20% Knockout serum supplement, 50 ng/mL fibroblast growth factor 2, 1× Insulin-Transferrin-Selenium, and 50 mg/mL 2-phospho-L-ascorbic acid. The cultures were refreshed with 2 mL of the above medium every 2 days until day 8. When colonies displayed iPSC morphology (between days 6 and 8 post-transfection), cells were fed with mTeSR1 medium (Stemcell Technologies, Vancouver, BC, Canada) supplemented with 0.25 mM sodium butyrate every 2 days until day 14. Colonies were picked manually on day 14–16 and cultured on Matrigel-coated dishes every 5–7 days until after 5 passages, when they were cryopreserved for further testing and profiling.

### 2.3. Culture Conditions for iPSCs

iPSCs were cultured and expanded on plates coated with Matrigel (Corning, New York, NY, USA, 354277) in either mTeSR1 or E8 (ThermoFisher Scientific, Waltham, MA, USA, A1517001) media. Cells were maintained at 37 °C with 5% CO_2_ with daily media changes, and split when cells reached 70–80% confluency (within 5–7 days of seeding). Any iPSC colonies with irregular borders, spontaneous differentiation, or transparent centers were manually removed prior to splitting. Cells were passaged by incubation in Gentle Cell Dissociation media (Stemcell Technologies, Vancouver, BC, Canada, 07174) for 4 min at 37 °C to obtain single cells, or RT for 6 min to obtain small aggregates of colonies. The following cell densities were used: 2 × 10^4^ cells/well in 24-well plates for immunocytochemistry, 2 × 10^4^/60 mm dish for daily morphology imaging, and 2 × 10^5^/well in 6-well plates for EB formation.

### 2.4. Crystal Violet Assay

For this assay, 4000 cells/well were plated in Matrigel-coated 96-well plates with mTeSR1 or E8 media. After 2, 4, or 6 days in culture, plates were rinsed with PBS to remove non-attached cells and fixed with 3.7% PFA for 5 min, before staining with 0.05% crystal violet (CV, Sigma, St. Louis, MO, USA, 46364) diluted in water for 30 min. The CV dye was thoroughly washed away using distilled water, and the plates were dried at RT. Once dried, the plates were imaged and quantification was performed [19] by adding 100 µL of methanol (Fisher Chemical, Waltham, MA, USA, 32435K7) to the wells to solubilize the CV, followed by measurement of the OD at 540 nm (OD540) with an EnSpire Multimode Plate Reader (Perkin Elmer, Waltham, MA, USA).

### 2.5. RNA Isolation, cDNA Synthesis, and qPCR Analysis

RNA was purified with a NucleoSpin RNA kit (Takara, Shiga, Japan, 740955) according to the manufacturer’s instructions. cDNA was generated using the iScript Reverse Transcription Supermix (BioRad, Hercules, CA, USA, 1708840). Quantitative real-time PCR was performed on the QuantStudio 5 Real-Time PCR System (Applied Biosystems, Waltham, MA, USA) using the primers listed in Supplementary Table S1. Raw data was processed using a custom Python script, available at https://github.com/neuroeddu/Auto-qPCR (accessed on 29 July 2020). The cycle threshold (CT) values for technical triplicates were tested for outliers. Relative gene expression was calculated by using the comparative CT method (ΔΔCT method), where the endogenous controls were GADPH or ACTB expression. The reference sample varied by experiment and is indicated in each plot.

### 2.6. Short Tandem Repeat (STR) Analysis

All newly generated iPSCs were authenticated through STR analysis with the GenePrint^®^ 10 System (Promega, Madison, WI, USA, B9510) at The Centre for Applied Genomics, the Hospital for Sick Children, Toronto. Briefly, genomic DNA from the iPSCs or the source material for the iPSCs, which in this case was PBMCs, was extracted with a Genomic DNA Mini Kit (Blood/Cultured Cell) (Geneaid, New TaiPei City, Taiwan, GB100). For this test, 10 ng of genomic DNA was mixed with GenePrint^®^ 10 primer pair mix to permit co-amplification and detection of ten human loci, including all ASN-0002-2011 loci (TH01, TPOX, vWA, CSF1PO, D16S539, D7S820, D13S317, D5S818) plus Amelogenin for gender identification and the mouse locus D21S11. These loci collectively provide a genetic profile, with a random match probability of 1 in 2.92 × 10^9^.

### 2.7. Karyotyping and Genomic Abnormalities Analysis

Genomic DNA was extracted with the Genomic DNA Mini Kit. Genomic integrity was detected with the hPSC Genetic Analysis Kit (Stemcell, 07550) according to the manufacturer’s instructions. Briefly, 5 ng of genomic DNA was mixed with a ROX reference dye and double-quenched probes tagged with 5-FAM. The probes represented eight common karyotypic abnormalities that have been reported to arise in hiPSCs: chr 1q, chr 8q, chr 10p, chr 12p, chr 17q, chr 18q, chr 20q, or chr Xp. Sample-probe mixes were analyzed on a QuantStudio 5 Real-Time PCR System (ThermoFisher Scientific). Copy numbers were analyzed using the ΔΔCt method. The results were normalized to the copy number of a control region in chr 4p [20]. For G-band karyotyping, iPSCs were cultured for 72 h until they attained 50–60% confluency, then were shipped live to the Wicell Cytogenetics Core (instructions provided by WiCell, Madison, WI, USA).

### 2.8. Three Germ Layer Differentiation Test

To form EBs, 2 wells of a 6-well plate containing 80–90% confluent iPSCs were dissociated into small clumps and cultured on low-attachment tissue plates in their preferred iPSC maintenance media (based on CV assays in Section 2.4). On day 7, EBs were transferred to Matrigel-coated plates and left to spontaneously differentiate for 14 days in DMEM media (Wisent, Saint-Jean-Baptiste, Canada, 319-005-CL) containing 20% FBS (Gibco, Waltham, MA, USA, 10091), 1% NEAA solution (Wisent, 321-011-EL), and 0.1 mM 2-mercaptoethanol (Gibco, 31350010) prior to fixation and immunocytochemistry.

To differentiate iPSCs into each of the three germ layers, cells were passaged and dissociated as described above for EB generation into single cells and cultured on Matrigel-coated plates with the STEMdiff Trilineage differentiation kit (according to the manufacturer’s instructions, Stemcell Technologies, 05230). Cells were harvested on the indicated day for gene expression analysis by qPCR.

### 2.9. Immunocytochemistry Analysis

Cells were fixed in 4% PFA/PBS at RT for 20 minutes, permeabilized with 0.2% Triton X-100/PBS for 10 min at RT, then blocked in 5% donkey serum, 1% BSA, and 0.05% Triton X-100/PBS for 2 h. Cells were incubated with primary antibodies in blocking buffer overnight at 4 °C. Secondary antibodies were applied for 2 h at RT, followed by Hoechst 33,342 nucleic acid counterstain for 5 min. Immunocytochemistry images were acquired using the automated Evos FL-Auto2 imaging system (ThermoFisher Scientific). Antibodies used for staining are listed in Supplementary Table S2. Images were quantified using custom ImageJ macros. The analysis scripts are available at https://github.com/neuroeddu/CellQ (accessed on 29 July 2020). The thresholds were determined visually by comparing five randomly sampled images.

### 2.10. Cortical Neuron Differentiation

Differentiation into cortical neurons was based on a protocol for EB formation combined with dual inhibition of SMAD [21,22], with modifications. Briefly, each iPSC line was cultured in its preferred media for 5–7 days, then dissociated into single cells to form EBs. The EBs were grown in a low-attachment plate for one week in DMEM/F12 supplemented with N2 and B27, in the presence of 10 μM SB431542, and 2 µM DMH1. On day 7, EBs were transferred to polyornithine- and laminin-coated plates to form rosettes in the same media. On day 14, rosettes were selected semi-manually and cultured as a monolayer on polyornithine- and laminin-coated plates to generate neural progenitor cells (NPCs) in DMEM/F12 supplemented with N2 and B27. NPCs were passaged at a 1:3 dilution every 5–7 days. Immunocytochemistry and qPCR analysis of NPCs were conducted at day 25. NPCs were next cultured in neurobasal medium, supplemented with N2 and B27, in the presence of 1 μg/mL laminin, 500 μM db-cAMP, 20 ng/mL BDNF, 20 ng/mL GDNF, 200 μM ascorbic acid, 100 nM Compound A, and 1 ng/mL TGF-β for differentiation into neurons. Immunocytochemistry and qPCR analysis of cortical neurons were conducted at day 56.

### 2.11. Data Visualization and Statistical Analysis

All data visualization plots were created in R using the ggplot2 graphical package. For qPCR quantification, the standard deviation (SD) values were calculated in Python. For enhanced visualization of the qPCR quantification in Section 3.5 and Supplementary Figure S4, the values were log transformed, and the SD was equivalently scaled. For image analysis, SD values were calculated in R. For all plots, variation is presented as mean ± SD. A minimum of two independent replicates for each experiment were performed.

## 3. Results

### 3.1. Validation of hiPSC Culture Conditions

**Figure 2:**
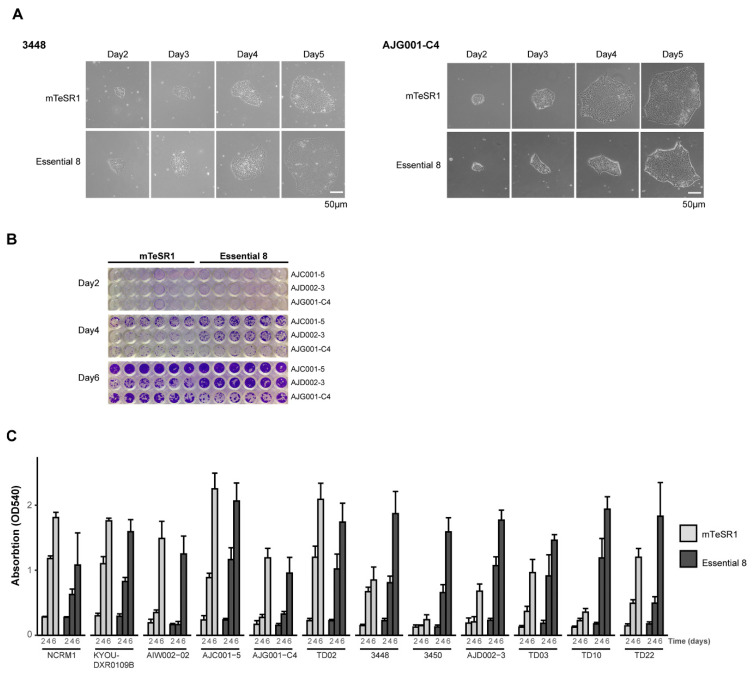
HiPSC growth and proliferation under different conditions. (**A**) Representative daily morphology of hiPSCs maintained in mTeSR1 or E8 media. Both cell lines show smooth-edged, tightly packed cells with a large nucleus-to-cytoplasm ratio. AJG001-C4 cells grow slightly better in mTeSR1 media, while 3448 proliferate better in E8. (**B**) CV assay of representative cell lines grown in mTeSR1 and E8. AJC001-5 grows well in both media. AJG001-C4 grows better in mTeSR1 media, while AJD002-3 cells proliferate best in E8. (**C**) Quantification of the hiPSCs’ survival and growth in different media. The CV stain was dissolved in methanol and optical density was measured at 540 nm (OD540). The mean and the standard deviation are from six replicates from two independent experiments.

**Figure 3:**
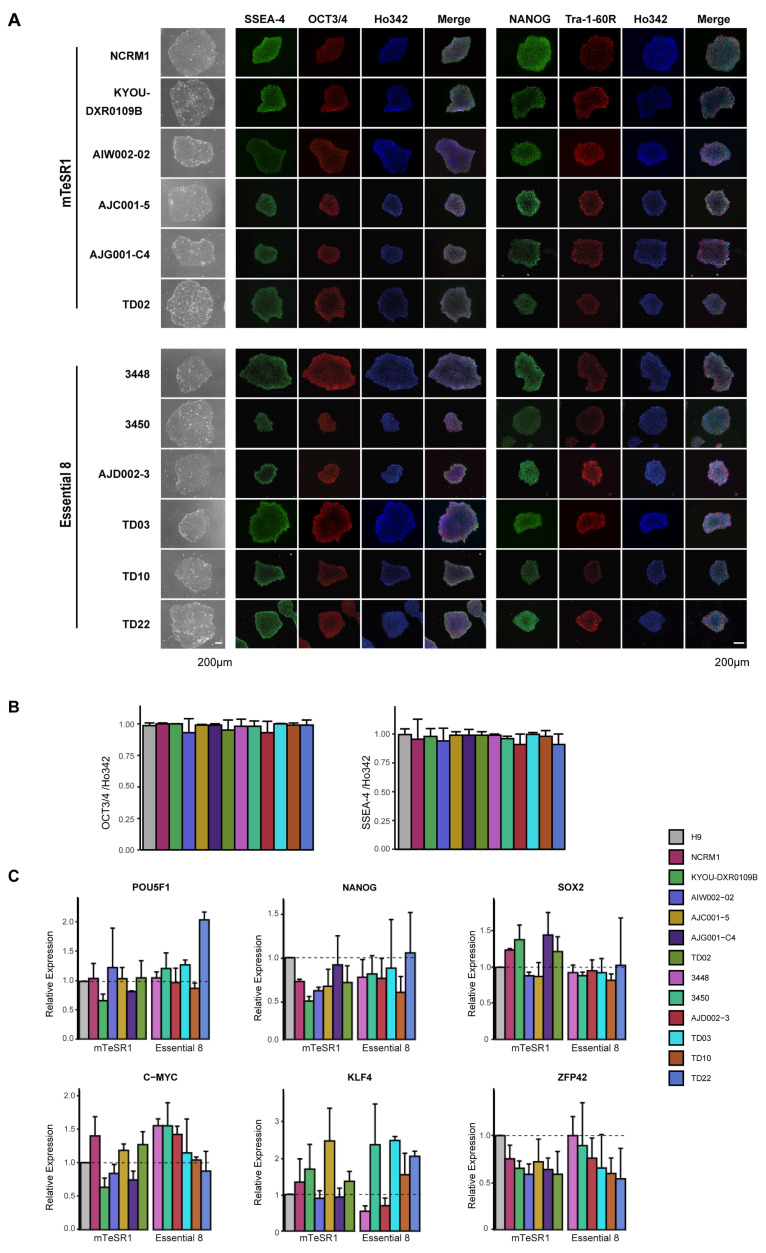
Expression of pluripotency markers in hiPSCs. (**A**) Phase contrast images and ICC for pluripotency markers SSEA-4, OCT3/4, NANOG, and Tra-1-60, with Ho342 nucleic acid counterstain. (**B**) Quantification of OCT3/4-positive cells or SSEA-4-positive cells shown in (**A**). (**C**) qPCR for mRNA expression of pluripotency genes POU5F1, NANOG, c-MYC, KLF4, SOX2, and ZFP42 in hiPSCs compared to H9 ESCs. mRNA expression in H9 was set as 1.0. The mean and SD are from technical triplicates from three independent experiments.

When working with any iPSC line, it is critical to first establish the optimal growth conditions for that cell line. As a first step in our workflow (Figure 1), our newly reprogrammed iPSCs and commercial iPSC lines (NCRM1-NIH and KYOU-DXR0109B-ATCC) were grown in parallel cultures with two distinct maintenance media: mTeSR1 or E8 (see cell line profiles in Table 1). Cells were seeded at an identical confluency and cultured in Matrigel-coated plates in either mTeSR1 or E8 media. We first examined cells 24 h after passaging for differences in their overall attachment, spontaneous differentiation, and morphology as they grew. All iPSC lines attached onto Matrigel-coated plates and presented with similar morphology when maintained in either medium. The condensed, round, diffuse, and irregular shape associated with iPSC colonies was observed across all cell lines. The colonies were smooth-edged, with tightly packed cells observed by phase contrast imaging (Figure 2A and Figure 3A). Rare incidences of spontaneous differentiation, in which the cells lost their pluripotency, could be observed in both media and across the different cell lines.

Although morphology is one indicator of iPSC quality, growth properties can vary from line to line, which can be influenced by the media. To detect differences in growth rates and adherence of cells over time, cells were fixed and stained with crystal violet (CV) (Figure 2B and Supplementary Figure S1), which demonstrated that the growth rate of all cell lines in each media was comparable at day 2. By day 4, three lines were observed to be growing at a faster rate in E8 compared to mTeSR1 (AJD002-3, 3450, and TD10; Figure 2C and Supplementary Figure S1). By day 6, the difference in growth rates was further pronounced for these lines, with 3448 and TD22 also demonstrating a preference for E8. Of note, the proliferation rate for AJG001-C4 was the slowest across the lines, growing at a comparable rate in both media (Figure 2C and Supplementary Figure S1). For our panel of iPSCs, variations in the growth rate between certain iPSCs were observed depending on the media conditions. Thus, in the rest of our assays, the cells were grown in their preferred media (Table 1). Taken together, daily morphological observations coupled with a CV assay enable a rapid and economical assessment of the growth conditions for a given cell line, ensuring that each iPSC line is cultured in optimal media for growth and expansion.

### 3.2. Characterization of iPSC Pluripotency

Next, we examined the pluripotency of each iPSC line (Figure 2). All iPSC lines tested expressed the pluripotency markers SSEA-4, OCT3/4, NANOG, and Tra-1-60R, in the same manner as the H9 ESC cell line, as determined by immunohistochemistry (ICC). The cell lines displayed no differences in the fluorescence intensity of these markers (Figure 3A). Notably, we found that over 90% of cells were OCT3/4- or SSEA-4-positive, demonstrating that the cells were maintained in a state of pluripotency with rare spontaneous differentiation (Figure 3B). The iPSCs were not only morphologically similar to each other and to embryonic stem cells (ESCs), but were also similar at a transcriptional level for a number of commonly observed pluripotency markers (OCT3/4, SOX2, NANOG) [23,24,25,26,27]. To examine the transcriptional profile of our lines, H9, an ESC line, was used as a reference line to normalize the expression of pluripotency genes relative to each of the control iPSC lines [28]. The expression of the Yamanaka factors (OCT3/4, SOX2, KlF4, and c-MYC) and two other widely tested pluripotent markers, NANOG and ZFP42 (Rex1) [29], were analyzed by qPCR (Figure 3C). We found that all iPSC lines expressed each of the pluripotency genes, and using a one-way ANOVA, we found that the expression level for each gene was comparable across all iPSC lines and the H9 ESC cell line, indicating that all iPSC lines met the threshold for being pluripotent. Combining the relative quantification of these six mRNAs, we observed significant correlations between each of the three independent sets of iPSC cultures (set1 vs. set2: R = 0.902, *p* = 5.60 × 10^−25^; Set1 vs. Set3: R = 0.726, *p* = 5.41 × 10^−14^; Set2 vs. Set3: R = 0.944. *p* = 1.25 × 10^−32^). From three independent biological replicates, these findings support the notion that our iPSC lines have a reproducible expression profile for pluripotency markers across each batch of cultures tested.

### 3.3. STR and Genomic Abnormality Testing of hiPSCs

The risk of cell misidentification and cross-contamination has plagued cell research [30,31]. In addition, hiPSCs which are generated and grown on a layer of mouse embryonic fibroblast (MEF) feeder cells are often at risk of cross-contamination with non-human rodent somatic cells [32]. For this reason, we conducted STR analysis for iPSC authentication. Our analysis demonstrated that the STR profile for each iPSC tested matched the parental cell line from which it was reprogrammed, and no rodent contamination was detected. As both AJD002-3 and TD03 were generated from the same donor, they displayed identical STR profiles relative to each other, and to somatic cells obtained from this donor (PBMC sample #3059) (Table 2).

**Figure 4:**
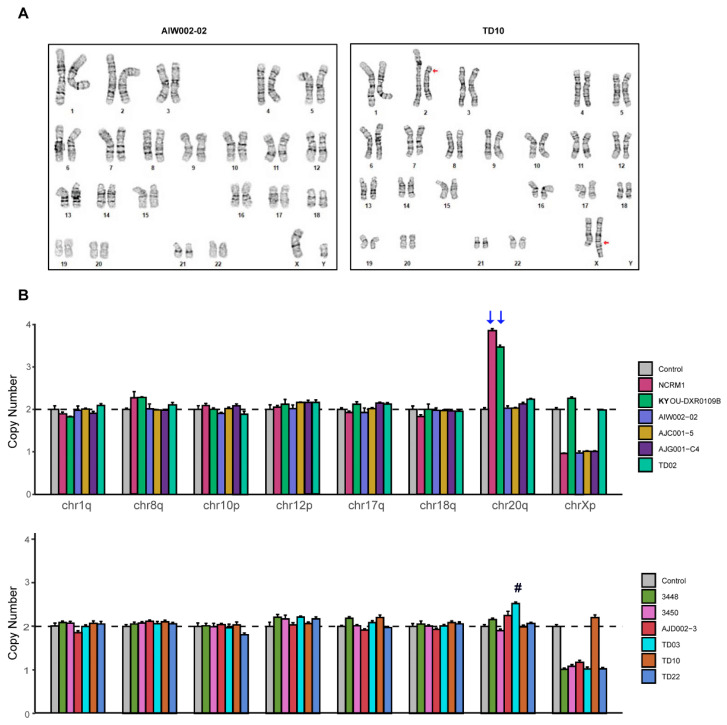
Genomic integrity analysis of hiPSCs. (**A**) Karyotyping and G-band analyses showing examples of one normal (left) and one abnormal hiPSC karyotype (right). An apparently balanced translocation between the long (q) arm of chromosome X and the short (p) arm of chromosome 2 is present in TD10 (red arrow). (**B**) qPCR-based genetic analysis to detect small chromosome abnormalities in all hiPSC lines. The copy numbers of chr1q, chr8q, chr10p, chr12p, chr17q, chr18q, chr20q, and chrXp were normalized to chr4p expression set at 2. Error bars show standard deviation from technical triplicates from two independent experiments. There are no abnormalities within critical regions in our cell lines, while NCRM1 and KYOU-DXR0109 have amplification of chr20q (indicated by blue arrows). TD03 shows a slight increase in chr20q (indicated with #), however this is not above the threshold of expected variation.

To assess chromosomal integrity of the iPSC lines, G-band karyotyping was performed. Our analysis showed that the majority of iPSCs tested had a normal 46, XY or 46, XX karyotype (Figure 4A and Supplementary Figure S2). However, we did detect a chromosomal anomaly in the TD10 line, which contained a translocation between the long (q) arm of chromosome X and the short (p) arm of chromosome 2. These abnormalities were confirmed to be a direct result of reprogramming, as follow-up G-band analysis of the parental PBMCs showed a normal karyotype (data not shown). This confirms that the chromosomal rearrangement in TD10 likely occurred during the reprogramming process and disqualifies this line from further use.

Although karyotyping by G-banding reveals both numerical and structural aberrations within chromosomes, the limited resolution of this method can only detect chromosomal aberrations greater than 5 Mb [14,33,34]. To test for commonly occurring genomic alterations, we used a qPCR-based genetic analysis kit to detect minimal critical hotspot regions within the genome that are frequently mutated during the reprogramming process and extended cell passaging, often conferring selective growth advantages to cells [35,36]. These regions include chr 1q, chr 8q, chr 10p, chr 12p, chr 17q, chr 18q, chr 20q, and chr Xp, which cover the majority of the reported abnormalities. In our newly generated iPSCs, we did not detect any increase or decrease in copy number outside the confidence interval (1.8 to 2.2), indicating that there were no abnormalities in the eight common hotspot zones tested (Figure 4B) [16,37,38,39,40], except for a moderate increase in the copy number of chr 20q in the TD03 line (Figure 4B, indicated with #). However, with both the commercial lines, NCRM1 and KYOU-DXR0109, an amplification in copy number on chromosome 20q was detected (Figure 4B, indicated with arrows), suggesting that this abnormality expanded with extensive cell passage. An amplification of chr20q was detected in high passage of 3450 (P18) compared to normal copy number at passage 5, further confirming how with extensive passaging, instabilities can arise (Supplementary Figure S2). Thus, it is imperative when working with any iPSC line to ensure that the line is profiled for the presence of genomic alterations which might affect the line’s growth and differentiation.

### 3.4. Differentiation of hiPSCs into Three Germ Layers

**Figure 5:**
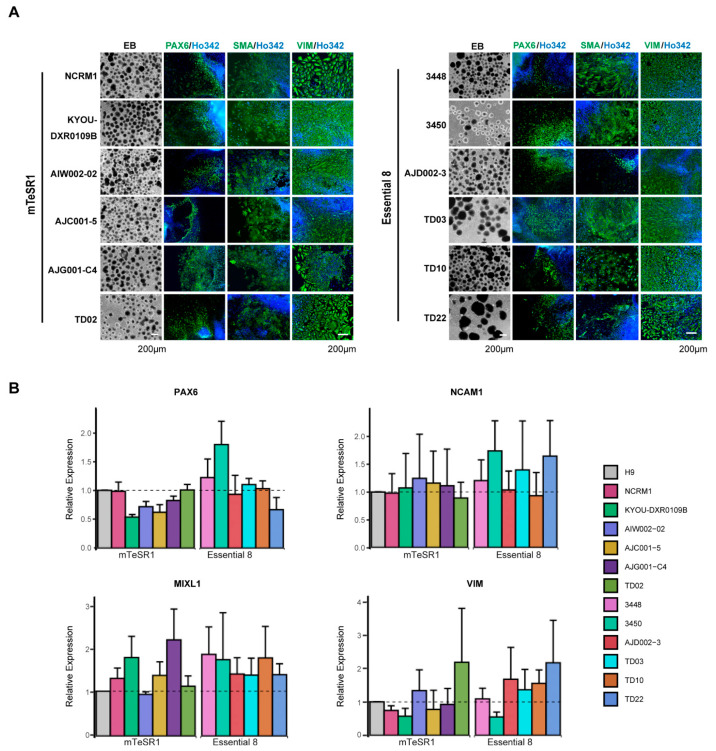
Differentiation of hiPSCs into three germ layers. (**A**) Representative phase contrast images of EB formation (left) and differentiation into three germ layers by ICC with the ectoderm (PAX6), mesoderm (SMA), and endoderm (VIM) markers (indicated above images). Nuclei are counterstained with Ho342. (**B**) qPCR for mRNA expression of three germ layer genes. Quantification of expression of the ectoderm (PAX6, NCAM1), mesoderm (MIXL1), and endoderm (VIM) markers in hiPSCs compared to H9 ESC. mRNA expression in H9 was set as 1.0. The mean and SD are from technical triplicates from three independent experiments.

One of the hallmarks of iPSCs is their ability to differentiate into almost any cell type of the three germ layers when provided with the appropriate developmental cues. To characterize the functional pluripotency of our newly generated iPSCs, we tested their ability to form EBs, in which cells spontaneously differentiate into each of the three embryonic germ layers. After one week, all iPSCs successfully formed EBs and no discernible differences in the relative size and total number of EBs formed was detected between lines. Following the formation of EBs in defined media, they were plated onto Matrigel-coated dishes in the presence of serum-containing media and cultured for 2 weeks. All iPSCs tested could differentiate into each of the three germ layers, as shown by positive immunostaining for the ectoderm marker PAX6, the mesoderm marker SMA, and the endoderm marker Vimentin (Figure 5A).

In parallel to image analysis, we also employed a qPCR-based assay for a faster, more quantitative assessment of functional pluripotency. We quantified the in vitro differentiation potential of our hiPSCs by measuring the relative expression of key genes that represent each of the three specific lineages, using the H9 ESC line as our control. As shown in Figure 5B, cells differentiated from iPSCs into each of the three lineage layers expressed ectodermal (PAX6 and NCAM), mesodermal (MIXL1), or endodermal (Vimentin) markers, depending on their given lineage. However, while all lines expressed each of these markers, variations were observed between lines, potentially indicating differing capabilities for each line to generate the different germ layers. For instance, 3450 expressed high levels of both Pax6 and MIXL1, while also expressing the lowest levels of Vimentin, indicating this line is likely best used to generate cells of an ectodermal or mesodermal lineage. In contrast, TD10 expressed all the lineage markers, indicating that the presence of the karyotypic abnormality had no effect on its ability to differentiate and form each of the three germ layers. ICC staining and qPCR findings confirmed that all iPSCs were capable of differentiation into each of the three germ layers. However, based on the expression analysis, the abilities of iPSCs to generate different cell types were variable, highlighting the need to choose iPSC lines carefully based on the cell types to be generated for downstream applications.

### 3.5. Differentiation of iPSCs into Cortical Neurons

**Figure 6:**
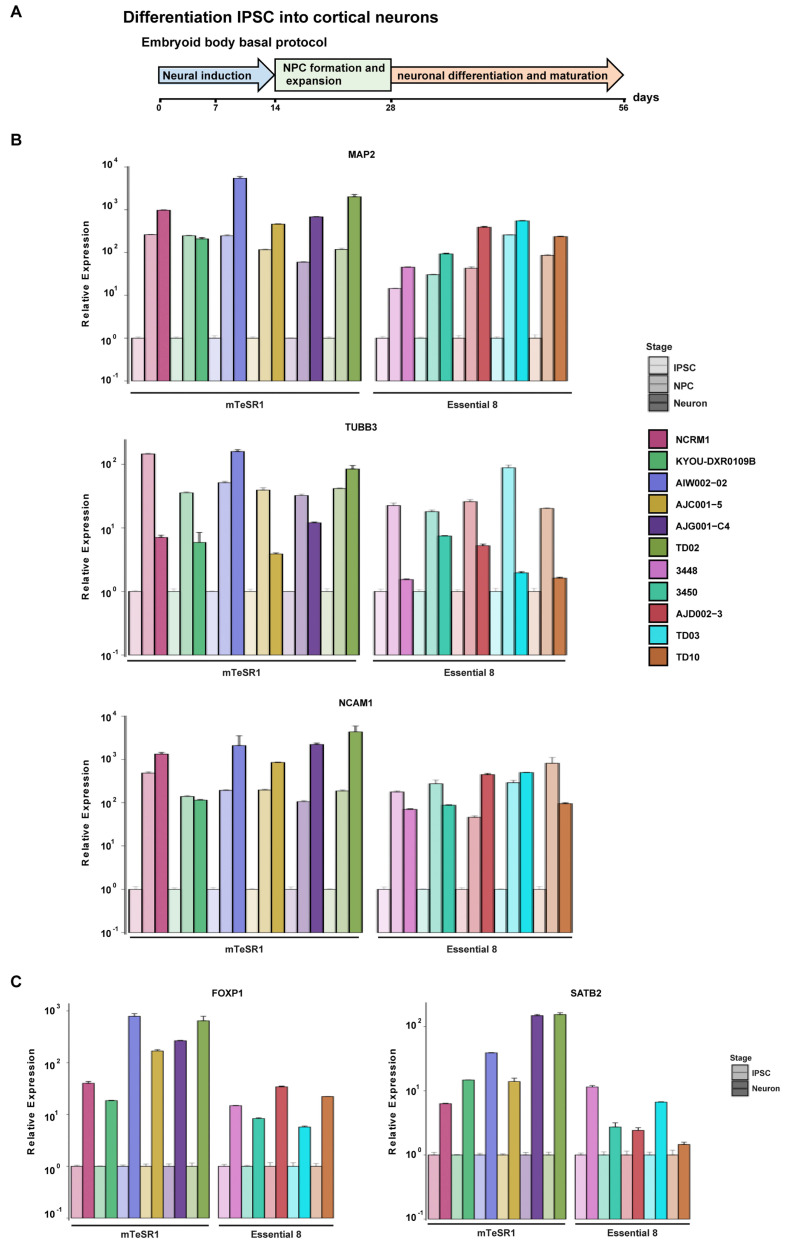
Differentiation of hiPSCs into forebrain cortical neurons. (**A**) Schematic representation of the in vitro differentiation protocol used to generate cortical neurons from hiPSCs. Boxes indicate differentiation state. This protocol was performed in 11 independent lines, with all lines performing similarly. (**B**) qPCR for mRNA expression levels of neuronal markers MAP2, NCAM1, and TUBB3 in NPC and 4-week-old differentiated cortical neurons compared to IPSCs. mRNA expression in iPSCs was set as 1.0. The mean and SD are from technical triplicates from two independent experiments. (**C**) Expression of cortical layer markers FOXP1 and SATB2 by qPCR analysis of 4-week-old differentiated cortical neurons compared to iPSCs. mRNA expression in iPSCs was set as 1.0. The mean and SD are from technical triplicates from two independent experiments.

Following trilineage analysis, we narrowed our focus down to one specific cell type from the ectodermal lineage: cortical neurons. To generate cortical neurons, we used a previously published dual SMAD inhibition protocol, with modifications [21,22]. Figure 6A shows the timeline schematic for the protocol, in which iPSCs were differentiated into neurons over 56 days. EBs were formed by dissociating iPSCs at day 0. After 14 days of neural induction, columnar neuroepithelium cells and typical neural tube-like rosettes appeared within all iPSC lines. Rosettes were dissociated and expanded into neural progenitor cells (NPCs). All characterization of NPCs was performed at day 25. We found that all iPSC lines generated NPCs, as demonstrated by positive immunostaining for the neural progenitor markers Nestin and SOX1, and maintained an ability to proliferate, as shown by the presence of the cell proliferation marker Ki67 (Supplementary Figure S3). Based on immunostaining quantification, approximately 60–80% of NPCs were Nestin-positive. In contrast, 10–25% of the cells from each line expressed SOX1 or Ki67 (Supplementary Figure S3B). Expression of the pluripotency markers (POU1F5 and NANOG; Supplementary Figure S4A) was significantly decreased at the NPC stage compared to the IPSCs (tOCT3/4 = 35,985; df = 10, *p* = 6.77 × 10^−42^; tNANOG = 960, df = 10, *p* = 3.71 × 10^−26^). These findings suggest that in spite of non-significant variation of mRNA levels between IPSC lines, they each significantly express markers of pluripotency We also quantified the mesodermal (MIXL1) and endodermal (AFP) markers. For both, we observed significant decreases in expression at the NPC stage compared to iPSCs (t_MIXL1_ = 3497, df _MIXL1_ = 10, p_MIXL1_ = 9 × 10^−32^; t_AFP_ = 96.33, df = 10, p_AFP_ = 3.56 × 10^−16^; Supplementary Figure S4B). The low levels of expression at the NPC stage were expected given that the cell type generated was of an ectodermal lineage (Supplementary Figure S4A,B). The expression of (1) the NPC marker Nestin, (2) dorsal forebrain progenitor markers (SLC1A3 and PAX6), and (3) the ventral forebrain progenitor marker ASCL1 were assessed by qPCR (Supplementary Figure S4C). Significant increases of these four mRNAs were observed at the NPC stage compared to iPSCs (t_NESTIN_ = −4.266, df_NESTIN_ = 10, p_NESTIN_ = 0.002; t_SLC1A3_: −5.405, df _SLC1A3_ = 10, p _SLC1A3_ = 3 × 10^−4^; t_PAX6_ = −2.429, df_PAX6_ = 10, p_PAX6_ = 0.035; t_ASCL1_ = −5.004, d_fASCL1_ = 10, p_ASCL1_ = 0.001). These findings support image-based findings that show that the iPSCs could be differentiated into NPCs [21,41].

**Figure 7:**
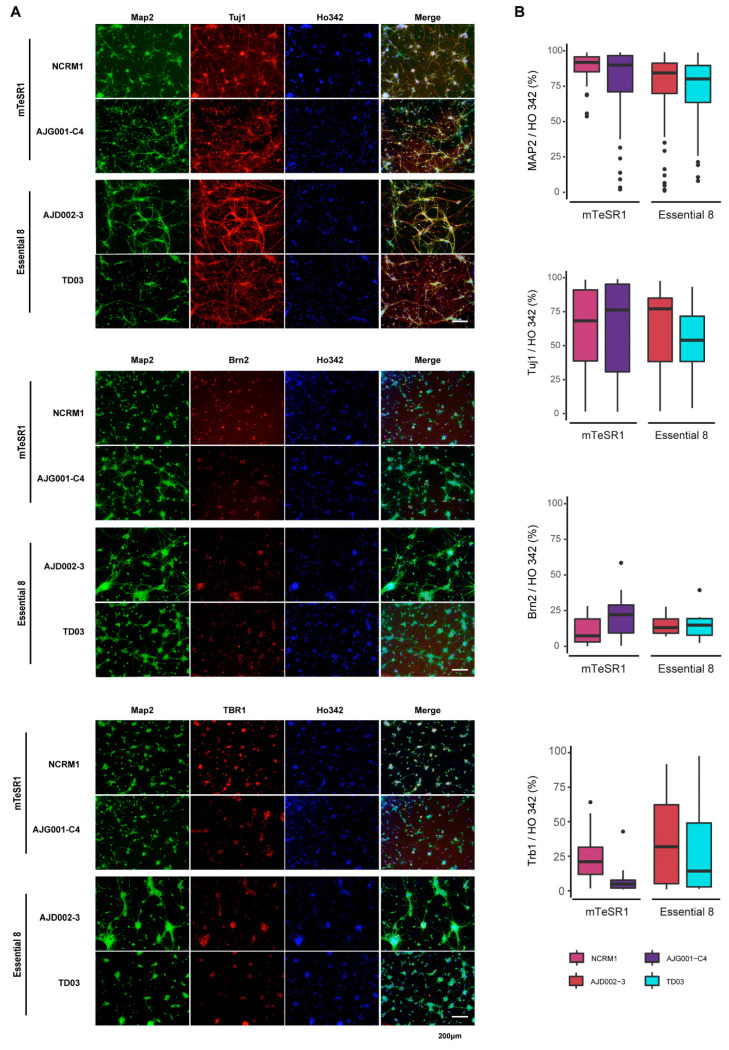
Immunocytochemistry staining of hiPSC-derived cortical neurons. (**A**) Representative images of ICC-stained cortical neurons from AJG001-C4, NCRM1, AJD002-3, and TD03 after 4 weeks of neuronal differentiation. Cells were stained with MAP2 and Tuj1 neuronal markers, and cortical neuron markers Brn2 and Tbr1. Nuclei were counterstained with Ho342. (**B**) Quantification of MAP2-, Tuj1-, Brn2-, or Tbr1-positive cells in (**A**).

NPCs generated from each line were subsequently differentiated into cortical neurons that were analyzed through a combination of qPCR and ICC analyses (Figure 6A). The expression levels of three neuronal markers (MAP2, NCAM1, and TUBB3) were quantified at the iPSC, NPC, and neuronal stages by qPCR. Using ANOVA for matched values, we observed significant changes in expression between stages for the three transcripts (D_MAP2_ = 4.1, df_MAP2_ = 2, p_MAP2_ = 0.032; D_NCAM1_ = 5.812, df_NCAM1_ = 2, p_NCAM1_ = 0.01; D_TUBB3_ = 4.518, df_TUBB3_ = 2, p_TUBB3_ = 0.024). These findings demonstrated that iPSC cells submitted to a cortical differentiation protocol acquire expression of neuronal markers, as observed at D56 (Figure 6B). Furthermore, upper layer (SATB2) or lower layer (FOXP1) cerebral cortex markers were also quantified. We found that both transcripts trended toward significant increases in neurons compared to iPSCs (t_FOXP1_ = −2.172, df_FOXP1_ = 10, p_FOXP1_ = 0.055; t_SATB2_ = df_SATB2_ = 10, p_SATB2_ = 0.069; Figure 6C). Based on quantification of the immunostained neurons, approximately 80–90% of cells expressed MAP2 and 60–80% of cells were Tuj1-positive neurons, varying from line to line (Figure 7 and Supplementary Figure S5). In contrast, the expression of the cortical neuron markers Brn2 or Tbr1 was much more variable (5–30%) across cell lines (Figure 7). Taken together, the iPSCs tested can generate neurons, although variations in morphology and expression of neuronal or cortical region markers were observed across lines.

## 4. Discussion

The routine use of iPSCs requires a constant supply of pluripotent, well-characterized, and quality-controlled cell stocks. However, without standardized quality control, experimental reproducibility with iPSCs can be compromised, making findings difficult to interpret [3,32,42]. Here, we established a workflow to monitor the morphology and proliferation of our newly generated iPSCs in two different media (mTeSR1 and E8). In parallel, we evaluated the genomic abnormalities, pluripotency, and differentiation potential of our lines. Based on these parameters, we can evaluate whether iPSCs can be used for further applications. However, this is just one iteration of a workflow, and its modular nature means additional quality-control tests can be added in further iterations. By focusing on cortical neuron differentiation from the panel of iPSCs, we can also better predict how variable cell lines are in their ability to generate cortical neurons with the same methods, while understanding how the neurons generated might differ from line to line, or from batch to batch within the same line.

Culture conditions can affect the quality, stability, and pluripotency of hiPSCs [9,15,43,44]. Today, commercially available media are widely used to culture hiPSCs [4,5,6,7,8]. We used CV staining [19], which is directly proportional to cell biomass, for our assays, which provides an affordable and straightforward method to quantify the proliferation of each cell line at different time points. However, other approaches can be performed to complement CV assays, from live cell imaging of iPSC growth rate, to fixed analysis for growth markers through immunocytometry and flow cytometry. While many of our lines grew in both media at comparable rates, some cell lines preferred one of the media used. Five cell lines grew faster in E8 (Figure 2B,C and Supplementary Figure S1), while other lines proliferated at a comparable rate in both media tested. The reason is not clear, but it has been demonstrated previously that the reduced composition of E8 (8 factors) can often elicit a faster growth rate [7]. It is also unclear why both the 3450 and TD10 cell lines grew well in E8, while their growth appeared to stall in mTeSR1. Interestingly, we found that cell lines derived from the same donor (AJC001-5 and AIW002-02, Table 1), albeit different cell types (PBMC vs. skin fibroblasts), tended to grow at comparable rates in either medium. Among lines generated from the same donor’s PBMCs (AJD002-3 and TD03), the reprogramming methods (Sendai CytoTune vs. episomal) did not appear to affect the preferred media. Thus, before working with any iPSC, it is imperative that culture conditions and media are optimized. Moreover, if a given iPSC line appears to have a poor growth rate with one media, it is recommended to test other media to ensure that this issue is due to the growth conditions and not the iPSC line itself.

Expression of pluripotency-associated markers is an important quality criterion for any iPSC line, otherwise the iPSCs cannot be differentiated into a cell type of interest. All iPSC lines in our study expressed the pluripotency markers SSEA-4, OCT3/4, NANOG, and TRA-1-60R in defined culture conditions (Figure 3A,B). We did find that OCT3/4 expression levels were approximately 15 times higher than those of SOX2 (Supplementary Table S3), consistent with previous studies, showing that this OCT3/4 high, SOX2 low stoichiometry is important not only in the early phase of reaching a fully reprogrammed state, but also in the late phase of iPSC maturation and maintaining pluripotency [45,46,47]. Further evidence has also demonstrated that OCT3/4 downregulates the downstream gene expression of NANOG, SPP1/OPN, SOX2, FBXO15, OTX2, and ZFP42/REX1 [48]. The expression levels of NANOG, ZFP42, and c-MYC in our study were approximately 50 times lower than those of OCT3/4, which is consistent with previous results from single-cell RNA-sequencing iPSC datasets [49,50].

A core feature of hiPSCs is their pluripotency, that is, the ability to differentiate into nearly any cell type of the three germ layers. The previous gold standard method to assess pluripotency of iPSCs was a teratoma assay, in which iPSCs were injected into immune-deficient mice to assess their ability to form teratomas [23,51]. However, this required the sacrifice of animals, and could be expensive and time-consuming, which led to the development of trilineage assays to assess the pluripotency of the cells [52]. One such approach is the in vitro formation of EBs, a commonly used method to assess the differentiation capability of a given iPSC [53,54]. All the iPSCs we tested had the potential to differentiate into each of the three germ layers, as shown by positive immunostaining for the previously reported ectoderm (PAX6), mesoderm (SMA), and endoderm (Vimentin) markers [55] (Figure 5A). However, while we successfully generated each of the three germ layers, the heterogeneous nature of the EBs resulted in inefficient and often variable differentiation of the three germ layers with each line. To further standardize our protocols, we used a commercial trilineage differentiation kit to perform parallel in vitro directed differentiation experiments for each germ layer. We also took advantage of a qPCR-based assay to enable a faster, more quantitative assessment of functional pluripotency, relative to the image-based approach of the EB trilineage test. Through this approach, we could quantify the in vitro differentiation potential of our iPSCs by measuring the relative expression of key genes that represent each of the three lineages. All the iPSCs expressed markers for each of the three germ layers, albeit at differing levels, with some lines expressing higher levels of one or more markers relative to each other (Figure 5B). Nevertheless, depending on the line, some consideration needs to be given as to whether it is the optimal iPSC line for generating a particular cell type of interest.

Numerous studies have demonstrated that both ESCs and iPSCs accumulate genomic abnormalities during long-term culturing. The presence of genetic variations in iPSCs has raised serious safety concerns for both patient interventions and basic research studies, hampering the advancement of novel iPSC-based therapies. G-banding was widely used for genetic evaluation [14], and upon karyotyping, the majority of iPSCs we tested maintained a normal 46, XY or 46, XX karyotype (Figure 4A and Supplementary Figure S2). However, we were able to detect a genomic anomaly in one of our control lines, TD10, which presented with an abnormal karyotype, that is, a translocation between the long (q) arm of chromosome X and the short (p) arm of chromosome 2. Yet, when compared to other iPSCs, this line appears within the normal range for other parameters, highlighting the importance of assessing each line for genomic abnormalities with multiple tests. One such test we used was a qPCR-based genetic analysis kit to detect minimal critical hotspot regions within the genome that can arise during the reprogramming process or confer selective growth advantages to a given cell line [35,36]. Using this analysis, which covers the majority of reported abnormalities, we did not detect any abnormalities in any of the hotspot zones with our newly generated iPSCs, although with extended passaging of the line 3450, an amplification in copy number on chromosome 20q was detected, similar to NCRM1 and KYOU-DXR0109 that had already been extensively passaged beyond 15+ passages (Figure 4B, labeled with an arrow, and Supplementary Figure S4B) [16,37,38,39,40]. These findings strongly suggest that it is critical to test lines for genomic abnormalities that can arise through reprogramming and prolonged cell passage. An additional quality-control test worth pursuing in future iPSC profiling is whole genome sequencing (WGS) [56,57]. With recent advances significantly reducing the cost of WGS, this technique could help detect low-frequency genetic alterations which would not be identified by conventional methods and add an extra layer of quality-control profiling to future workflows.

Moving beyond a broad trilineage test, we next tested our lines for their ability to form one specific cell type of interest, that is, cortical neurons. Prior studies have shown that dual SMAD inhibitors synergistically destabilized the activin- and NANOG-mediated pluripotency network [58], suppressed BMP-induced meso-/endodermal fate differentiation [59,60], and promoted neuralization of the primitive ectoderm by BMP inhibition [21]. qPCR analysis of gene expression in NPCs revealed that treatment with SB431542 and DMH1 induced a dramatic loss of the pluripotency markers POU1F5 and NANOG. Expression of the mesoderm (MIXL10) and endodermal (AFP) markers was also significantly decreased in all cell lines (Supplementary Figure S4A,B), as previously reported [21]. While we found increased PAX6 and Nestin expression levels in iPSC-derived NPCs (Supplementary Figure S4C), an expected result, we also aimed to determine if the variation of expression of iPSC and NPC markers was comparable to other studies. Maussion and colleagues compared expression levels of genes involved in neurodevelopmental processes, in post-mortem brain tissue, in NPCs and in iPSC-derived neurons [61]. Interestingly, many of the transcripts examined were included in our analysis. We compared the amplitude of the differences in POU1F5 and NANOG expression between iPSCs and NPCs in this earlier paper with our results. Using a paired t-test, decreased expression of POU1F5 and NANOG in NPCs compared to iPSCs was not significantly different in the two studies (t = −1.003; df = 1, *p* = 0.499). Reciprocally, we also compared the variation of expression for PAX6 and Nestin between iPSCs and NPCs in both studies and found that the increased expression of PAX6 and Nestin at the NPC stage was not significantly different (t = −0.734; df = 1; *p* = 0.597). These comparisons support the premise that the data presented in our pipeline corroborate observations made in a previous study regarding the change of iPSC and NPC expression markers with neuronal induction. For cortical neurons, 80–90% of cells expressed the neuronal marker MAP2 and 60–80% were Tuj1-positive neurons, although this varied from line to line. Consistent with published findings, these neurons also demonstrated a cortical layer identity, albeit one that also varied across lines. Based on ICC and qPCR results, ~5–25% of neurons from the different iPSCs tested expressed Brn2, a marker of upper-layer L2–L5 cortical neurons previously shown to regulate general migration mechanism [62]. The upper-layer identity has been further demonstrated by elevated expression of SATB2, another transcription factor found in cortical L2–L5 neurons [63]. Further evidence of this variability in neuronal identity was shown for the post-mitotic corticothalamic neuronal marker TBR1, which could be found in anywhere from 5% to 30% of neurons. The expression of Foxp1 in cortical neurons also supported the lower-layer feature [63]. Taken together, our findings indicated no preference for a specific layer in the generation of cortical neurons by the dual SMAD inhibition EB method, yet the expression levels were highly variable across iPSCs. The variability does not come from the differentiation protocol, as all the iPSCs were differentiated under the same conditions. This variability may not simply be a direct result of distinct genetic background differences, since variations in differentiation were also detected between AIW002-02, AJC001-5, and AJG001-C4, which share the same genetic background.

## 5. Conclusions

The reprogramming of somatic cells into iPSCs opens up the possibility of modeling human diseases and developing new therapeutics. Using human iPSC-derived cells for preclinical and clinical research will require a constant supply of well-characterized pluripotent cell lines. Thus, in this study, we established a workflow to monitor the growth and morphology of newly generated iPSCs in two different media. We also performed a comprehensive phenotyping of the iPSC lines through growth rate profiling, testing of genome integrity, analysis of pluripotency capacity, and tests on each of the iPSCs to form each of the three germ layers, with a particular focus on cortical neurons of the ectodermal lineage. From these studies, we demonstrated that our newly generated iPSC lines share common hallmarks, yet can vary in their growth rate or ability to differentiate into other cell types. Given our findings, it is imperative that each new iPSC line be evaluated thoroughly before using it in downstream applications, while ensuring the line can be used to generate the cell types of interest for a given research application. With these parameters in mind, the workflow outlined here will help to streamline work processes and offers the potential to add new tests as technologies evolve, to ensure researchers employ iPSCs of the highest quality for experimental reproducibility and robustness.

## Figures and Tables

**Table 1:** Overview of hiPSCs.

Cell Line	Donor	Cell Type	Donor Age	Sex ^1^	Cell Source	Reprogramming	Media ^2^
H9		ESC	NA	F	WiCell		mTeSR1
NCRM1	A	cord blood	NA	M	NIH	Episomal	mTeSR1
KYOU-DXR0109B	B	fibroblast	36	F	ATCC	Sendai virus	mTeSR1
AIW002-02	C	PBMC	37	M	The Neuro	Sendai virus	mTeSR1
AJC001-5	C	fibroblast	37	M	The Neuro	Sendai virus	mTeSR1
AJG001-C4	C	PBMC	37	M	The Neuro	Episomal	mTeSR1
TD02	D	PBMC	48	F	The Neuro	Episomal	mTeSR1
3448	E	PBMC	48	M	The Neuro	Episomal	E8
3450	F	PBMC	37	M	The Neuro	Episomal	E8
AJD002-3	G	PBMC	44	M	The Neuro	Sendai virus	E8
TD03	G	PBMC	44	M	The Neuro	Episomal	E8
TD10	H	PBMC	64	F	The Neuro	Episomal	E8
TD22	I	PBMC	59	M	The Neuro	Episomal	E8

^1^ M, male; F, female. ^2^ Optimal media for cell growth. The passage numbers of NEURO hiPSC lines are 8–12; H9: P30 + P5; NCRM1: P50 + P11; KYOU-DXR0109B: +P3 after cell had been purchased from ATCC, no passage information on product datasheet.

**Table 2:** Representative STR profiling of AJD002-3 and TD03 for authentication.

	3059 PBMC		AJD002-3 iPSC		TD03 iPSC	
Marker	Allele 1	Allele 2	Allele 1	Allele 2	Allele 1	Allele 2
AMEL	X	Y	X	Y	X	Y
CSF1PO	11	12	11	12	11	12
D13S317	9	11	9	11	9	11
D16S539	9	11	9	11	9	11
D21S11	28	30	28	30	28	30
D5S818	9	11	9	11	9	11
D7S820	12	12	12	12	12	12
TH01	6	9	6	9	6	9
TPOX	8	9	8	9	8	9
vWA	14	15	14	15	14	15

## Data Availability

Not applicable.

## Supplementary Materials

The following are available online at https://www.mdpi.com/article/10.3390/mps4030050/s1, Figure S1: HiPSC growth and proliferation profile in mTeSR1 or Essential 8 media; Figure S2: HiPSCs maintain a normal karyotype; Figure S3: Characterization of hiPSC-derived NPCs; Figure S4: Gene expression profiles in neural progenitor cells; Figure S5: Comparison of cortical neuron differentiation across two different differentiation tests; Table S1: Primers used in qPCR experiments; Table S2: Key resources; Table S3: Quantification of pluripotency gene expression in hiPSCs by qPCR analysis.

## Abbreviations

5-FAM	5-carboxyfluorescein
CV	crystal violet
E8	Essential 8 media
ESC	embryoid body
ESC	embryonic stem cell
hiPSC	human iPSC
Ho342	Hoechst 33342
ICC	immunocytochemistry
iPSC	induced pluripotent stem cell
NE	neuroepithelial
NEAA	non-essential amino acids
NPC	neural progenitor cell
PBMC	peripheral blood mononuclear cells
PFA	paraformaldehyde
QC	quality control
RT	room temperature
