# Supplementary material for: A Multistep Workflow to Evaluate Newly Generated iPSCs and Their Ability to Generate Different Cell Types
Source: Methods Protoc. 2021 Jul 19;4(3):50. doi: 10.3390/mps4030050 (PMC8293472; doi:10.3390/mps4030050)
Supplement: Supplementary file 1 [file mps-04-00050-s001.zip › mps-1215148-SI.pdf]

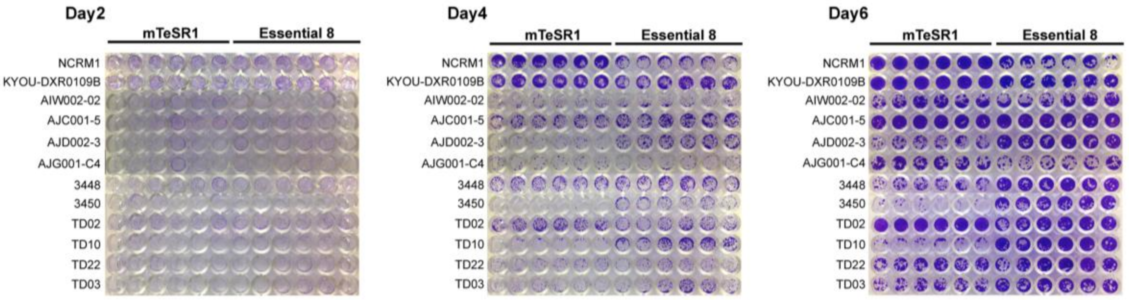

Figure S1. HiPSC growth and proliferation profile in mTeSR1 or Essential 8 media.

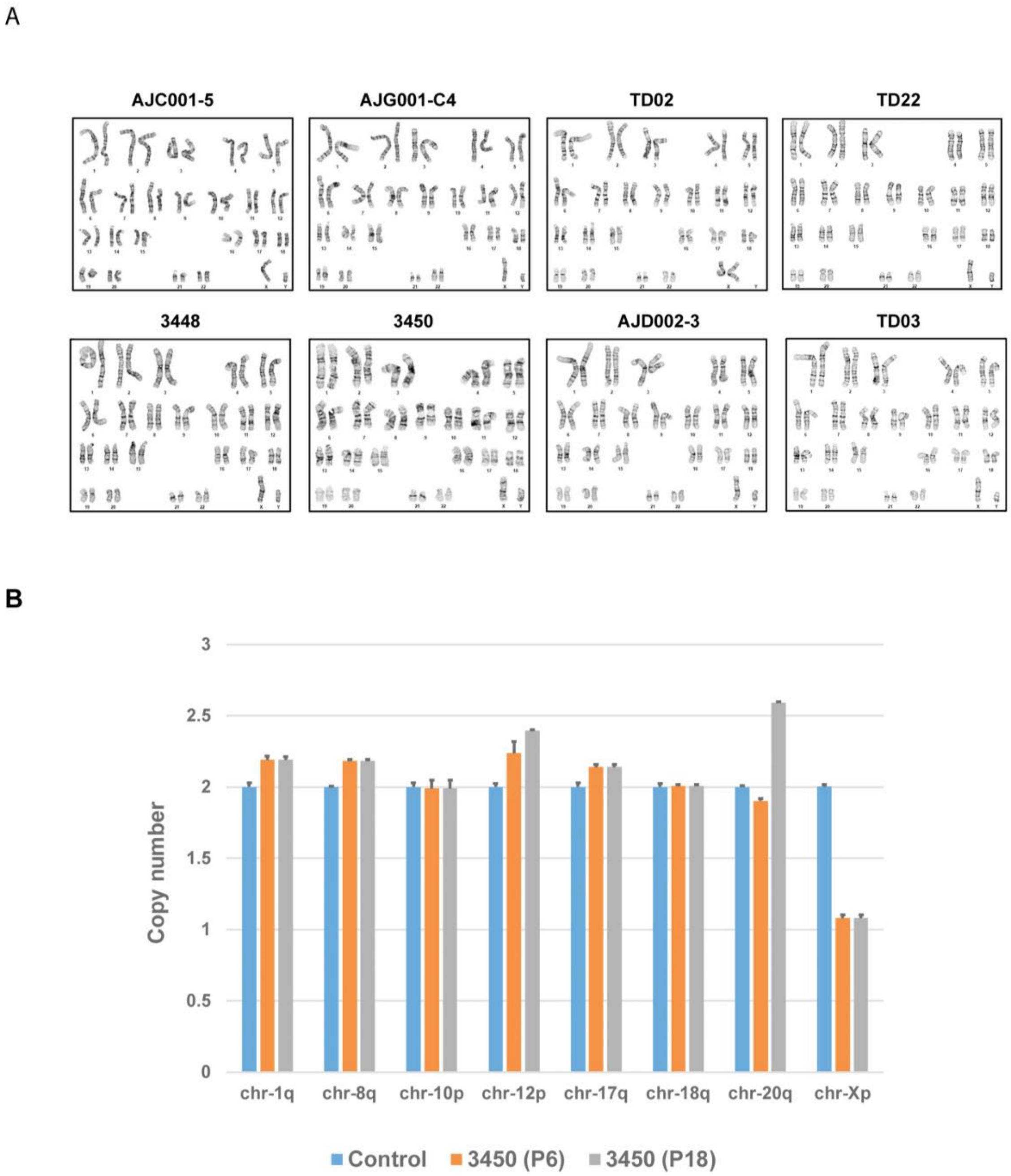

Figure S2. HiPSCs maintain a normal karyotype.

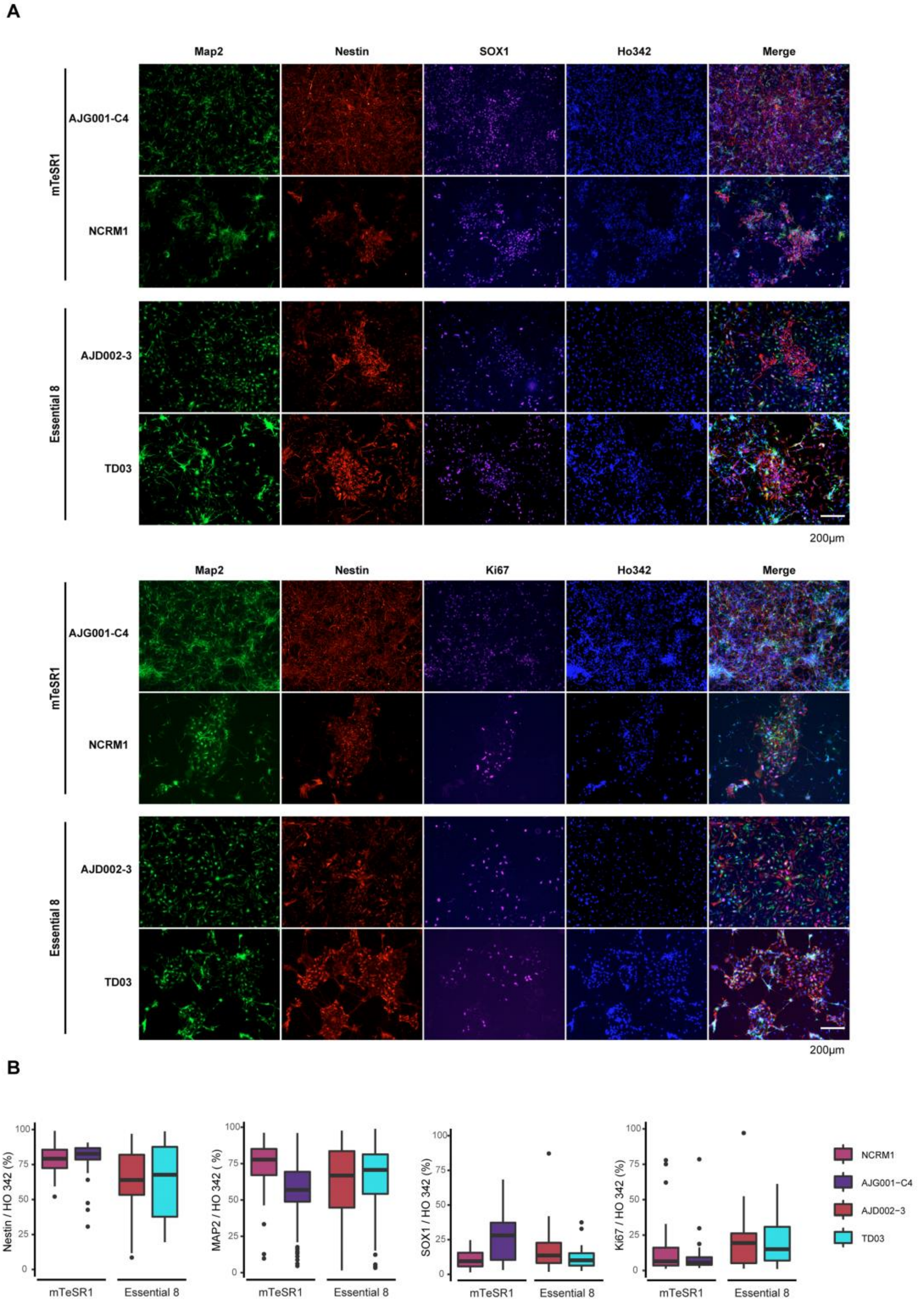

Figure S3. Characterization of hiPSC-derived NPCs.

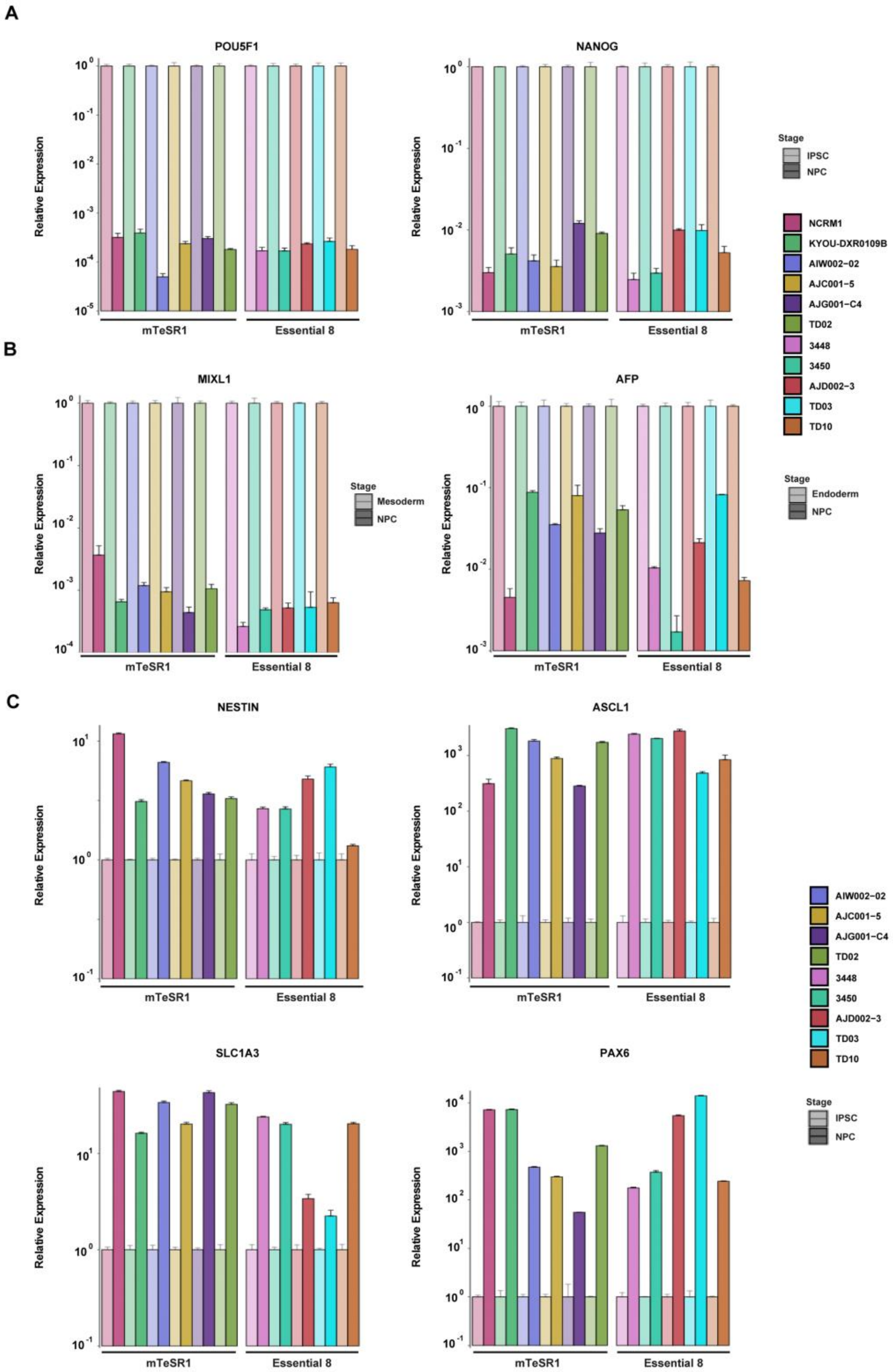

Figure S4. Gene expression profiles in neural progenitor cells.

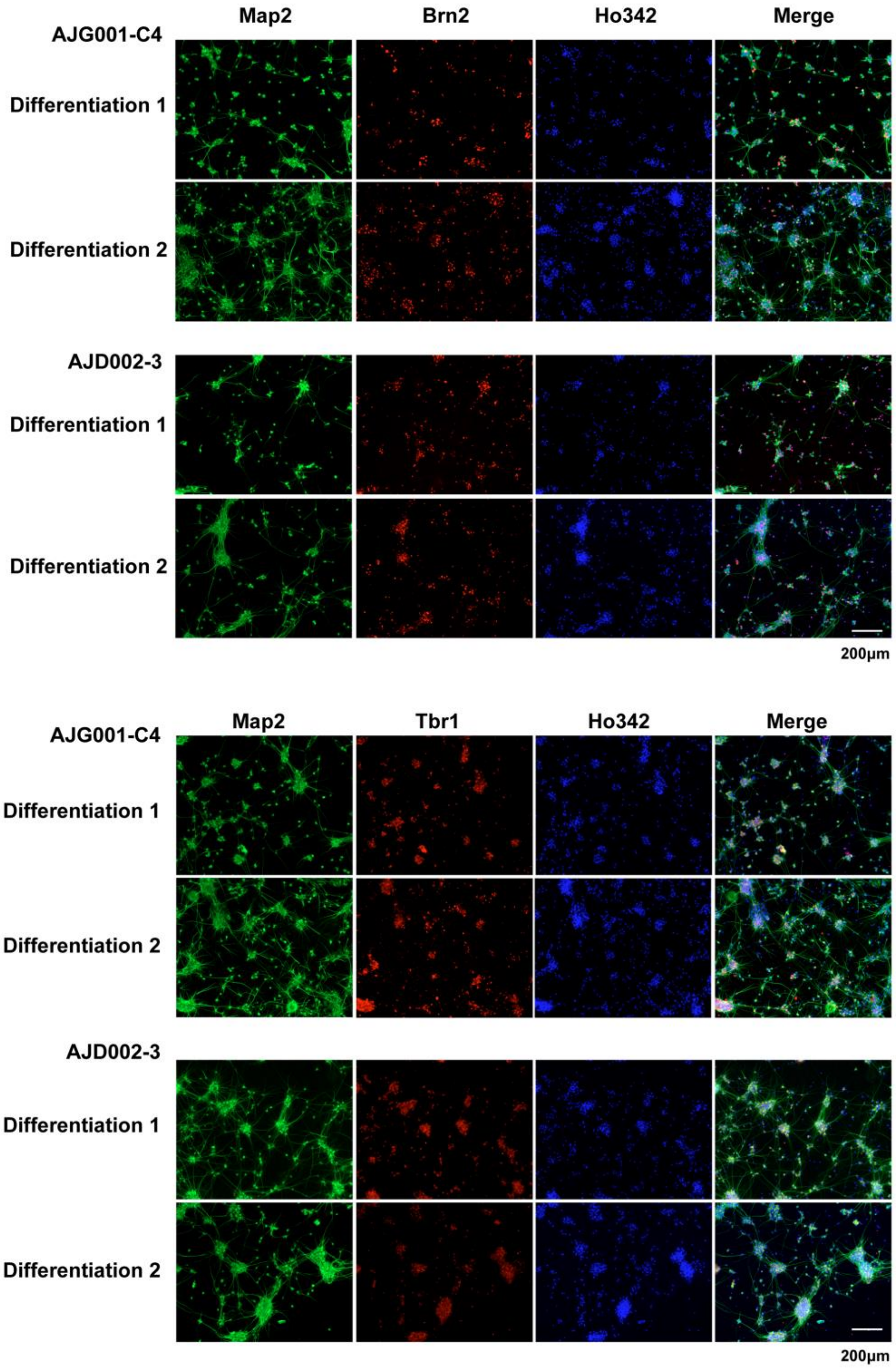

Figure S5. Comparison of cortical neuron differentiation across two different differentiation tests.

Table S1. Primers used in qPCR experiments.

Gene ID	Gene Name	Accession number	Assay ID
ACTB	Actin, beta	NM_001101.3	Hs01060665_g1
AFP	Alpha fetoprotein	NM_001134.2	Hs01040598_m1
ASCL1	Achaete-scute family bHLH transcription factor 1	NM_004316.3	Hs00269932_m1
MYC	v-myc avian myelocytomatosis viral oncogene homolog	NM_002467.4	Hs00153408_m1
GAPDH	Glyceraldehyde-3-phosphate dehydrogenase	NM_001256788.2	Hs02786624_g1
KLF4	Kruppel like factor 4	NM_001314052.1	Hs00358836_m1
MAP2	Microtubule associated protein 2	NM_001039538.1	Hs00258900_m1
MIXL1	Mix paired-like homeobox	NM_031944.2	Hs00430824_g1
NANOG	NANOG homeobox	NM_001297698.1	Hs02387400_g1
NCAM1	Neural cell adhesion molecule 1	NM_000615.6	Hs00941830_m1
NES	Nestin	NM_006617.1	Hs04187831_g1
PAX6	Paired box 6	NM_000280.4	Hs01088114_m1
POU5F1 (OCT3/4)	POU class 5 homeobox 1	NM_001173531.2	Hs04260367_gH
SLC1A3	Solute carrier family 1 member 3	NM_001166695.2	Hs00904823_g1
SOX2	SRY-box 2	NM_003106.3	Hs04234836_s1
TUBB3	Tubulin beta 3 class III	NM_001197181.1	Hs00801390_s1
VIM	Vimentin	NM_003380.3	Hs00958111_m1
ZFP42	ZFP42 zinc finger protein	NM_001304358.1	Hs00399279_m1

Table S2. Overview of antibodies.

Antibody	SOURCE	IDENTIFIER
Rabbit polyclonal anti-Brn2/POU3F2	Cell Signaling	Cat#12137; RRID: AB_2797827
Mouse monoclonal anti-CD44	BD Biosciences	Cat#550392; RRID: AB_2074674
Mouse monoclonal anti-Ki-67	BD Biosciences	Cat#556003; RRID: AB_396287
Chicken polyclonal anti-MAP2	EnCor Biotechnology	Cat#CPCA-MAP2; RRID: AB_2138173
Rabbit polyclonal anti-Nanog	Abcam	Cat#ab21624; RRID: AB_446437
Rabbit polyclonal anti-Nestin	Abcam	Cat#ab92391; RRID: AB_10561437
Goat polyclonal anti-Oct-3/4(N-19)	Santa Cruz Biotechnology	Cat#sc-8628; RRID: AB_653551
Mouse monoclonal anti-PAX6	DSHB	Cat#AB_528427; RRID: AB_528427
Rabbit polyclonal anti-SMA1	Abcam	Cat#ab5694; RRID: AB_2223021
Goat polyclonal anti-SOX1	R&D Systems	Cat#AF3369; RRID: AB_2239879
Rabbit polyclonal anti-SOX9	Millipore	Cat#AB5535; RRID: AB_2239761
Mouse monoclonal anti-SSEA-4(813-70)	Santa Cruz Biotechnology	Cat#sc-21704; RRID: AB_628289
Rabbit monoclonal anti-TBR1	Abcam	Cat#ab31940; RRID: AB_2200219
Mouse monoclonal anti-TRA-1-60R	Stemcell Technologies	Cat#60064; RRID: AB_2686905
Rabbit monoclonal anti-Vimentin	Abcam	Cat#ab92547; RRID: AB_10562134
Mouse monoclonal anti-Tubulin- βIII	Millipore	Cat#MAB5564; RRID: AB_11212768
Hoechst 33342	ThermoFisher Scientific	H3570

Table S3. Quantification of pluripotency gene expression in hiPSCs by qPCR analysis (mean ± SEM of relative gene expression, normalized to GAPDH and ACTIN).

Cell line	POU5F1	SOX2	NANOG	c-MYC	ZFP42	KLF4
**H9**	2.144±0.131	0.1704±0.0276	0.0708±0.0173	0.0434±0.0022	0.0644±0.0127	0.000186±0.000047
**NCRM1**	2.189±0.401	0.2114±0.1178	0.0505±0.0123	0.0597±0.0092	0.0508±0.0153	0.000291±0.000179
**KYOU-DXR0109B**	1.446±0.313	0.2225±0.0874	0.0351±0.0119	0.0281±0.0130	0.0439±0.0014	0.000259±0.000084
**AIW002-02**	2.723±1.539	0.1526±0.0299	0.041±0.0081	0.0407±0.014	0.0688±0.0016	0.000173±0.000073
**AJC001-5**	2.194±0.271	0.1383±0.0202	0.042±0.0134	0.0406±0.0089	0.0363±0.0020	0.000493±0.0027
**AJG001-C4**	1.763±0.114	0.2646±0.076	0.0525±0.0105	0.0437±0.0158	0.0513±0.0024	0.000162±0.000054
**TD02**	2.199±0.486	0.196±0.0179	0.0431±0.0102	0.0325±0.0066	0.0329±0.0024	0.000259±0.000099
**3448**	2.248±0.078	0.1526±0.0182	0.0465±0.002	0.0596±0.0044	0.0618±0.0053	0.000099±0.000039
**3450**	2.553±0.416	0.1483±0.0189	0.0498±0.005	0.0462±0.0133	0.0433±0.0014	0.000493±0.000316
**AJD002-3**	2.035±0.396	0.1527±0.0121	0.0454±0.0047	0.0442±0.0056	0.0461±0.0014	0.000126±0.000054
**TD03**	2.636±0.006	0.1377±0.0733	0.0364±0.0206	0.0467±0.0018	0.0312±0.0104	0.000431±0.000017
**TD10**	1.863±0.072	0.1365±0.0209	0.0376±0.0138	0.0347±0.0069	0.0318±0.0027	0.000328±0.000176
**TD22**	4.222±0.345	0.1237±0.0051	0.0494±0.012	0.0250±0.0105	0.025±0.0015	0.000333±0.000104
